# Lysophospholipids and Their G-Coupled Protein Signaling in Alzheimer’s Disease: From Physiological Performance to Pathological Impairment

**DOI:** 10.3389/fnmol.2020.00058

**Published:** 2020-04-15

**Authors:** Yining Hao, Min Guo, Yiwei Feng, Qiang Dong, Mei Cui

**Affiliations:** ^1^Department of Neurology, Huashan Hospital, Fudan University, Shanghai, China; ^2^Department of Neurology, Huashan Hospital, State Key Laboratory of Medical Neurobiology and MOE Frontiers Center for Brain Science, Fudan University, Shanghai, China

**Keywords:** lysophospholipids, lysophosphatidic acid (LPA), sphingosine-1-phosphate (S1P), G-protein coupled receptor (GPCR), Alzheimer’s disease (AD)

## Abstract

Lysophospholipids (LPLs) are bioactive signaling lipids that are generated from phospholipase-mediated hydrolyzation of membrane phospholipids (PLs) and sphingolipids (SLs). Lysophosphatidic acid (LPA) and sphingosine-1-phosphate (S1P) are two of the best-characterized LPLs which mediate a variety of cellular physiological responses *via* specific G-protein coupled receptor (GPCR) mediated signaling pathways. Considerable evidence now demonstrates the crucial role of LPA and S1P in neurodegenerative diseases, especially in Alzheimer’s disease (AD). Dysfunction of LPA and S1P metabolism can lead to aberrant accumulation of amyloid-β (Aβ) peptides, the formation of neurofibrillary tangles (NFTs), neuroinflammation and ultimately neuronal death. Summarizing LPA and S1P signaling profile may aid in profound health and pathological processes. In the current review, we will introduce the metabolism as well as the physiological roles of LPA and S1P in maintaining the normal functions of the nervous system. Given these pivotal functions, we will further discuss the role of dysregulation of LPA and S1P in promoting AD pathogenesis.

## Introduction

The human brain is highly rich in lipids, which account for approximately 60%–70% of its dry weight (Yung et al., 2015). These lipids can be generally divided into two major clusters: lipids which act as structural components and lipids which function as signaling molecules, according to their functional heterogeneity (Yung et al., 2015). Structural lipids are the primary components of cellular membranes. They form dynamic functional rafts and serve as platforms for the membrane proteins (Simons and Ikonen, 1997). Meanwhile, the signaling lipids, which are considered to be the bioactive lipids, exert their effects by binding to specific receptors acting in a signal transduction mechanism (Choi and Chun, 2013). Lysophospholipids (LPLs) are metabolic intermediates generated *via* the active hydrolyzation of phospholipase on membrane phospholipids (PLs) and sphingolipids (SLs). Two major bioactive lipid derivatives are playing a crucial role in various cellular physiological processes as well as pathological events, the well-characterized lysophosphatidic acid (LPA) and sphingosine-1-phosphate (S1P; Li et al., 2016). LPA and S1P function mainly as extracellular mediators by activating cognate cell surface G-protein coupled receptors (GPCRs) and stimulate intracellular responses through different signaling transduction pathways. Sophisticated and well-balanced modulation of LPA and S1P metabolism has been suggested to be critical in the developing and mature nervous system (Choi and Chun, 2013).

Most neurodegenerative diseases are accompanied by changes in both the composition and metabolism of LPLs (Wang and Bieberich, 2018). Reoccurring evidence has indicated that loss homeostatic LPA and S1P metabolism may act as a co-participator in the pathogenesis of multiple neurodegenerative disorders, particularly in Alzheimer’s disease (AD; Ghasemi et al., 2016; Wang and Bieberich, 2018). Indeed, both LPA and S1P have been demonstrated to participate in the generation of neuropathological hallmarks that characterize AD by binding to their G protein-coupled LPLs receptors (LPL-GPCRs). Dysfunction of LPA and S1P metabolism results in aberrant amyloid-β peptide (Aβ) aggregation (Shi et al., 2013), neurofibrillary tangle (NFT) formation (Sayas et al., 2002b), neuroinflammation (Awada et al., 2014; Kwon et al., 2018) and ultimately neuronal apoptosis (Robinson, 2015). In this review, we summarized the metabolism of LPA and S1P as well as their GPCRs cell signaling, with emphasis on the physiological role of LPA and S1P in the nervous system, and their underlying crosstalk with AD pathogenesis.

## Metabolism of Cellular LPA and S1P

### LPA Metabolism

LPA, also known as 1-acyl 2-hydroxylglycerol 3-phosphate, is an autocoid PL that is formed on-demand and functions near to the location of its production (Li et al., 2016). The generation of bioactive LPA requires phospholipase A2 (PLA2) mediated cleavage of a membrane PL, for example, the phosphatidylcholine. In this instance, arachidonic acid (AA) is generated in addition to a LPL, such as lysophosphatidylcholine (LPC). The latter then acts as the substrate for producing LPA by a dual-function ectoenzyme named autotaxin (ATX), while AA is further converted to pro-inflammatory mediators. ATX, also known as the ectonucleotide pyrophosphatase/phosphodiesterase-2 (ENPP2), is a soluble enzyme mainly found in plasma and cerebrospinal fluid (CSF; Herr et al., 2020). Aberrant ATX expression and malfunction in the autotaxin–LPA (ATX–LPA) axis have been suggested to promote the initiation and progression of AD pathology (Ramesh et al., 2018; Herr et al., 2020).

### S1P Metabolism

S1P levels in human tissues are under sophisticated regulation with two bioactive enzymes; sphingosine kinase (SphK), which is related to S1P biosynthesis, and sphingosine-1-phosphate lyase (SPL), which governs S1P degradation. Physiologically, membrane sphingomyelin is degraded to ceramide, which is subsequently converted to sphingosine by the enzyme ceramidase. Sphingosine is then phosphorylated to S1P by highly regulated SphKs in various cellular compartments (Spiegel and Milstien, 2003; Santos and Lynch, 2015). Sphingosine kinases (SphKs) are a cluster of evolutionarily conserved lipid kinases, which modulate S1P production. There are two isoforms of SphK, known as SphK1 and SphK2, of which the subcellular localizations are consistent with the compartmentalization and biological effects of S1P (Chan and Pitson, 2013). SphK1 is found localized in the cytoplasm and is activated only when recruited to the cell membrane, while SphK2, as a membrane related lipid kinase, mainly concentrates on cellular organelles, such as the nucleus, mitochondria and endoplasmic reticulum (ER; Neubauer and Pitson, 2013). The discrepancy of the subcellular localization between SphK1 and SphK2 indicates the different biological functions they mediate (Spiegel and Milstien, 2003; Alvarez et al., 2010). SphK1 generated S1P signaling requires the re-localization of SphK1 from the cytosol to the plasma membrane, thus stimulating cell migration, proliferation, and survival (Zhu et al., 2010; Chan and Pitson, 2013; Gassowska et al., 2014). By contrast, the biological effects of SphK2 are more complicated. SphK2 has been implicated in the inhibition of DNA synthesis in the nuclei (Hait et al., 2009). It has also been proved to promote apoptosis by interacting with Bax and Bak in mitochondria (Chipuk et al., 2012). Furthermore, in serum deprivation conditions, localization of SphK2 in the ER has been suggested to activate a “salvage” pathway named “sphingolipid rheostat” by promoting the generation of pro-apoptotic ceramide instead of protective S1P (Neubauer and Pitson, 2013).

## G-Coupled Protein Receptors (GPCRs) of LPA and S1P in The Cell Signaling

Both LPA and S1P are cell membrane-derived bioactive LPLs. They function *via* the 7-transmembrane GPCRs on the cell surface to activate multiple downstream signaling cascades (Choi and Chun, 2013; Li et al., 2016). There are four classes of heterotrimeric G proteins involved in the LPL mediated GPCR signals: G_s_, G_q/11_, G_i_, and G_12/13_. By binding to specific GPCRs, LPA and S1P mediate physiological processes as well as pathological events within the nervous system (Choi and Chun, 2013).

Six LPA receptors (LPARs) have been identified : LPA1-LPA6, with genes named LPAR1-LPAR6 (human) and Lpar1-Lpar6 (non-human; Kihara et al., 2014; Yung et al., 2014, 2015). LPARs have been found to function in neurogenesis and brain development (Castilla-Ortega et al., 2011), neurodifferentiation (Fukushima et al., 2002b; Spohr et al., 2008), neural network formation and morphogenesis (Furuta et al., 2012; Roza et al., 2019), neuroplasticity (Fujiwara et al., 2003; Rhee et al., 2006) and glia cell modulation (Shano et al., 2008; Awada et al., 2014).

Similarly, five S1P receptors (S1PR1-5) belonging to the Edg family engage in the regulation of cellular biological events through G_q_, G_i_, G_12/13_, and Rho proteins (Li et al., 2016; Czubowicz et al., 2019). S1P with specific high-affinity S1PRs, have multiple ancillary roles in the regulation of oxidative stress (Sinha et al., 2013; Pyszko and Strosznajder, 2014), apoptosis (Edsall et al., 1997; Ghasemi et al., 2016), mitochondrial dysfunction (Chan and Pitson, 2013; Santos and Lynch, 2015; Ghasemi et al., 2016), autophagy (Moruno Manchon et al., 2015) and neuronal development (Mizugishi et al., 2005; Hait et al., 2014; Ghasemi et al., 2016). It should also be noted that S1P employs several mechanisms to act as a bioactive participant in subcellular compartmentalization, thus indicating a complicated biological effect of S1P and S1P mediated intracellular transduction (Maceyka and Spiegel, 2014). Identification of LPA and S1P associated G-protein-coupled LPL receptors, signaling mechanisms, and biological and pathological functions in the central nervous system (CNS) are summarized in Table 1.

**Table 1:** Signaling mechanism, G-protein-coupled lysophospholipids receptors, and their identified physiological and pathological functions.

Receptors	G proteins	Signaling pathways	Biological functions	Pathological events
**LPARs**				- Neurodegeneration (Choi and Chun, 2013; Yung et al., 2015).
LPA1	G_i/o_	PLC↑, MAPK↑, PI3K/Akt↑, Rac↑ AC↓, cAMP↓	- Neuronal development (Contos et al., 2000; Fukushima et al., 2002a; Kihara et al., 2014; Suckau et al., 2019); - Neurogenesis of adult hippocampus (Fukushima et al., 2002a);	- Neuropathic pain (Choi et al., 2010); - Ischemic stroke (Savitz et al., 2006; Kimura et al., 2008); - Schizophrenia (Cunningham et al., 2006; Musazzi et al., 2011); - Behavioral dysfunctions (Santin et al., 2009); - Seizure (Elmes et al., 2004).
	G_12/13_	Rho↑	- Survival and differentiation of neural progenitor cells (NPCs; Chun et al., 2002; Fukushima et al., 2002a; Yung et al., 2014);	
	G_q/11_	PLC↑, (Ca^2+^)_i_↑	- Modulation of adult hippocampus neuroplasticity (García-Morales et al., 2015; Peñalver et al., 2017; Roza et al., 2019); - Negative feedback of microglia induced inflammation (Awada et al., 2014; Kwon et al., 2018).
LPA2	G_i/o_	PLC↑, MAPK↑, PI3K/Akt↑, AC↓, cAMP↓	- Neuronal development (Suckau et al., 2019); - Neuronal differentiation (Spohr et al., 2008; E Spohr et al., 2011);	- Seizure (Elmes et al., 2004; Trimbuch et al., 2009).
	G_12/13_	Rho↑	- Survival and differentiation of neural progenitor cells (NPCs; Chun et al., 2002);
	G_q/11_	PLC↑, (Ca^2+^)_i_↑	- Neuronal hyperexcitability and neuro-plasticity (Broggini et al., 2010; Coiro et al., 2014);	
LPA3	G_i/o_	PLC↑, MAPK↑, PI3K/Akt↑, AC↓, cAMP↓	- Neurite branching (Furuta et al., 2012; Roza et al., 2019);	- Neuropathic pain (Ma et al., 2009).
	G_q/11_	PLC↑, (Ca^2+^)_i_↑		
LPA4	G_i/o_	PLC↑, MAPK↑, PI3K/Akt↑, Rac↑ AC↓	- Morphogenesis and migration of newborn cortical neurons (Kurabayashi et al., 2018);
	G_12/13_	Rho↑		
	G_q/11_	PLC↑, (Ca^2+^)_i_↑		
	G_s_	AC↑, cAMP↑		
LPA5	G_12/13_	Rho↑	- Promotion of microglia migratory response and shift to a pro-inflammatory phenotype (Plastira et al., 2016, 2017).	- Neuropathic pain (Lin et al., 2012).
	G_q/11_	PLC↑, (Ca^2+^)_i_↑		
LPA6	G_12/13_	Rho↑		
**S1PRs**				- Neurodegeneration (Chakrabarti et al., 2016; Karunakaran and van Echten-Deckert, 2017).
S1P1	G_i/o_	PLC↑, PI3K/Akt↑, Rac↑, ERK ↑	- Neurogenesis and brain development (Mizugishi et al., 2005) - Neurite extension (Toman et al., 2004); - Inhibition of cortical glutamatergic neurotransmission (Sim-Selley et al., 2009);	- Neuropathic pain (Sheng et al., 2014; Boyette-Davis et al., 2015); - Multiple sclerosis (Choi et al., 2011); - Spinal cord injury (Goldshmit et al., 2010);
S1P2	G_i/o_	PLC↑, PI3K/Akt↑, Rac↑, ERK ↑	- Neurite extension (Toman et al., 2004); - Regulation of neuronal excitability (MacLennan et al., 2001).	- Seizure (MacLennan et al., 2001); - Ischemic stroke (Kimura et al., 2008); - Behavioral dysfunctions (Akahoshi et al., 2011);
	G_12/13_	Rho↑, Rac↓		
	G_q/11_	PLC↑, (Ca^2+^)_i_↑		
S1P3	G_i/o_	PLC↑, PI3K/Akt↑, Rac↑, ERK ↑		
	G_12/13_	Rho↑, Rac↓		
	G_q/11_	PLC↑, (Ca^2+^)_i_↑		
S1P4	G_i/o_	PLC↑, PI3K/Akt↑, Rac↑, ERK ↑		
	G_12/13_	Rho↑, Rac↓		
S1P5	G_i/o_	PLC↑, PI3K/Akt↑, Rac↑, ERK ↑	- Inhibition of oligodendrocyte progenitor cells (OPCs) migration (Novgorodov et al., 2007).	- Multiple sclerosis (Miron et al., 2010).
	G_12/13_	Rho↑, Rac↓		

## Physiological Roles of LPA in The Nervous System

LPA acts as a functional bioactive lipid, exerting its effects by binding to six cognate cell surface GPCRs termed LPARs (LPA1-LPA6). The importance of LPA signaling has been stressed by accumulating evidence about their involvement in the regulation of cell proliferation, survival, migration, cytoskeletal change, and cell to cell interaction in the nervous system (Choi and Chun, 2013; Yung et al., 2014). In this section, the LPA-mediated biological functions in the CNS will be discussed with detail.

### LPA and Brain Development

It has been proven that LPA acts as a crucial regulator of cortical development and neurogenesis. Embryos with mutations of ATX, the key ectoenzyme that modulates LPA biosynthesis, are unable to achieve cranial neural tube closure. In this regard, using expression analysis of neural marker genes, Koike et al. (2011) have demonstrated reduced lateral expansion of the rostral forebrain and impaired midbrain-hindbrain boundary in ATX mutant embryos. Also, animal experiments suggested defects in neural tube development in Enpp−/− mutant mice (Moolenaar et al., 2013). In the same way, LPARs are also required in the modulation of cortical development since the LPAR genes are dynamically regulated in time and space-dependent manner at various developmental stages in the mouse brain and hippocampal primary neurons (Suckau et al., 2019).

Deficiency of LPA1 has been shown to reduce cortical width as well as cerebral wall thickness, thus leading to smaller brain size, and more severely, 50% perinatal lethality (Contos et al., 2000). Consistently, similar work also indicated disabled migration, morphological changes, proliferation, and differentiation in LPA1-null neuronal cells (Contos et al., 2000; Kihara et al., 2014), suggesting a crucial role of LPA1 in modulating neurogenesis-related events. Moreover, it is also well evidenced that LPA4 remodels the actin cytoskeleton and promotes microtubule formation in neurons, thus leading to bipolar morphogenesis and radial migration of newborn cortical neurons (Fukushima et al., 2002b; Kihara et al., 2014; Kurabayashi et al., 2018).

Several lines of evidence are available which indicate that the LPA mediated cortical development and neurogenesis are realized by modulating neural progenitor cells (NPCs). LPA has been shown to mediate NPC ionic conductance alterations (Yung et al., 2014). It must be emphasized that, among all of the subtypes of LPAR, LPA1 and LPA2 are two of the best-described receptors that mediate the LPA-induced survival and differentiation of NPCs (Chun et al., 2002). Besides the mentioned roles, LPA1 and LPA2, along with LPA6, have been characterized as key players in regulating astrocyte and oligodendrocyte development, synapse formation, maturation and stabilization (Suckau et al., 2019). Collectively, this evidence illustrates the regulatory role of LPA and its receptors in brain development.

### LPA and Neuronal Differentiation

The first step of neuronal differentiation requires the formation of neurites, through a key process known as neuritogenesis. An array of evidence indicates that LPA has a controlling role in neurite growth within the embryonic brain (Hecht et al., 1996), and collective observations have suggested LPA-elicited neurite retraction, cell rounding, and growth cone collapse in various neuronal cell lines (Tigyi and Miledi, 1992). Satoh et al have reported that LPA-induced neurite retraction can be prevented by inhibiting the Rho/Rho-kinase pathway (Satoh et al., 2011), thus suggesting an underlying signaling pathway. Consistently, fasudil, a Rho-kinase inhibitor that acts as an efficacious treatment of ischemic brain infarction by improving hemodynamic function as well as preventing inflammatory response (Satoh et al., 2008), has recently been demonstrated to prohibit neurite retraction (Satoh et al., 2008). Moreover, research has also shown that LPA can activate the small GTPase Rho, which will in turn contract the cytoskeleton in neurite retraction (Tigyi and Miledi, 1992; Savitz et al., 2006; Kimura et al., 2008). These results are in line with the study conducted by Yamazaki et al. (2008) which showed that LPA induces growth cone collapse and neurite retraction *via* the G_12/13_-RhoA-GSK3β pathway. Indeed, LPA has been identified to primarily influence GSK3β-mediated rearrangement of microfilaments (MFs) and polymerization-dependent microtubules (MTs). Such interactions between MF-MT have been indicated to regulate LPA-induced neurite outgrowth (Fukushima and Morita, 2006; Fukushima et al., 2011). In this regard, conclusions can be drawn that LPA-mediated G_12/13_ activation increases RhoA GTPase activity, thus leading to the upregulation of GSK-3, downstream disorganization of MTs, growth cone collapse as well as the retraction of neurites (Sayas et al., 1999).

In addition to the LPA induced Rho/Rho-kinase pathway activation of cytoskeletal rearrangement, it has also been suggested that LPA could also stimulate another form of actin rearrangement by modulating intracellular calcium. It has been well evidenced that activation of LPARs can stimulate phospholipase C (PLC), thus leading to a temporary release of calcium from the ER. Furthermore, it has also been validated that LPA can directly induce Ca^2+^ influx from the extracellular fluid (Jang et al., 2014). Fukushima et al. (2002a) have demonstrated two distinct types of actin remodeling induced by LPA within neuroblasts (Fukushima et al., 2002a). By binding to the same LPAR, activation of the Rho/Rho-kinase pathway induced actin polymerization and the subsequent neurite retraction, whereas elevation of the intracellular calcium concentration led to α-actinin depolymerization and resulted in the loss of membrane ruffling (Fukushima et al., 2002a). This LPA-PLC-Ca^2+^ mediated cytoskeletal rearrangement could be significantly reversed by decreasing intracellular calcium (Fukushima et al., 2002a).

Besides the mentioned role of LPA in modulating cytoskeletal rearrangement, Spohr et al. (2008) have also indicated that LPA could regulate neuronal differentiation by affecting other cellular events such as cell fate determination and maturation in direct and indirect ways. Similar results are also demonstrated by Dottori et al. (2008) that high concentrations of LPA can inhibit neuronal differentiation by either affecting cell cycle or increasing cell death.

### LPA and Neural Morphogenesis

Another important biological function of LPA lies in the regulation of neural morphogenesis, which involves the modulation of axons and neurites branching. There are two ways in which LPA mediates axon and neurite branch formation: direct and indirect. The former requires a novel signaling transduction pathway involving LPA3, G_q_, and Rnd2, while the indirect mechanism involves the small GTPase family. Furuta et al. (2012) have detected inhibition of neurite branching when both G_α i_ and G_α q_ were restrained. Besides the mentioned roles of Rho GTPase in regulating GSK3β-mediated cytoskeleton rearrangement, it has also been shown to modulate the axon’s response to neurotrophins. There has been validated evidence showing that the small GTPase family members such as Rho, Rac and Cdc42 are involved in the regulation of neurite outgrowth as well as neurite morphology (Govek et al., 2005). Govek et al. (2005) have observed that in the embryonic trigeminal cells, the Rho GTPases acted in a crucial role in neurotrophin-mediated axonal differentiation. They went on to further show that in the differentiation of axons and dendrites, Rac, another member of the small GTPase family, activates the response of trigeminal cells to neurotrophins (Yamazaki et al., 2008). In addition to these effects, the small GTPases, Rac and Rho have also been implicated in altering microglial morphology by regulating Kir2.1 channels (Muessel et al., 2013).

### LPA and Neuroplasticity

The LPAR LPA1 is widely expressed in human hippocampal progenitor cells (Castilla-Ortega et al., 2011), and several lines of evidence have indicated a regulatory role of LPA1 in promoting synaptic modifications in adult hippocampal neurons (Fujiwara et al., 2003; Rhee et al., 2006; Castilla-Ortega et al., 2011). Further evidence identified that the LPAR genes are under dynamic regulation throughout mouse brain development, modulating synaptic plasticity in a temporal- and spatial-dependent manner (Suckau et al., 2019). Roza et al. (2019) have proposed the underlying mechanism by which LPAR modulates synaptic plasticity. They detected presynaptic glutamate release when stimulating LPAR localized in the presynaptic terminals, whereas excessive LPA stimulation was found to cause seizures (Roza et al., 2019). These results are in line with the observation that LPA modulates the excitatory as well as the inhibitory synapses to regulate synaptic strength and neuronal activity (García-Morales et al., 2015). It has been demonstrated that LPA functions as a dual regulatory factor in modulating synaptic excitability. High levels of LPA have been shown to lessen the size of available vesicle pools at glutamatergic pre-synaptic neurons, thus providing negative feed-back to prohibit the transmission at excitatory synaptic terminals. Nevertheless, in the presence of low concentrations of LPA, inhibitory postsynaptic receptors, namely gamma-aminobutyric acid receptor type A (GABAAR), were internalized to restrain transmission at inhibitory synaptic terminals, thereby briefly increasing the synaptic excitability (García-Morales et al., 2015; Roza et al., 2019). In line with these findings, it is believed that LPA plays a crucial role in modulating glutamatergic transmission in the nervous system. Accordingly, LPA is also demonstrated to induce the phosphorylation of NMDA receptors, thus regulating the synaptic plasticity that is associated with learning and memory (Roza et al., 2019). Direct evidence is published showing that LPA triggers the phosphorylation of NMDA receptors and mediates the Ca^2+^ influx in the postsynaptic terminals, leading to either long-term potentiation or long-term depression at the synapses of both excitatory and inhibitory neurons (Roza et al., 2019).

In addition to the functional plasticity regulated by LPA, it has also been shown that the LPARs play a vital role in modulating the structural plasticity of neurons. Roza et al. (2019) have demonstrated that inhibition of LPA1 mediates the downregulation of both glutaminase isoform (GLS) and active matrix-metalloproteinase 9 (MMP-9) in the cerebral cortex and hippocampus. This downregulation thereby results in an alteration in the morphology of glutamatergic pyramidal cells to an immature phenotype, as well as cognitive and memory deficiency (Roza et al., 2019). Moreover, this phenomenon has also been well illustrated by Peñalver et al. (2017) who have shown that silencing of LPA signaling affected the expression of the biosynthesis of glutamate isoenzyme and induced the dendritic spines to differentiate towards a less mature phenotype. In line with these results, it is plausible to assume that LPA signaling contributes to the regulation of cognitive and memory-related neuroplasticity at a molecular level.

Plasticity-related gene 1 (PRG-1) is a member of the integrin family, which acts as a co-factor with LPA in modulating glutamate neurotransmission. It has been shown that the post-synaptic deficiency of PGR-1 prohibits LPA from entering the post-synaptic membrane, thereby leading to the accumulation of LPA in the synaptic cleft (Vogt et al., 2016). Thus, an increased level of LPA in the synaptic gap has been shown to have two complementary mechanisms which result in increased glutamate concentration in the synaptic cleft. First, the increased LPA level has been shown to stimulate pre-synaptical LPA2 and induce the release of glutamate (Trimbuch et al., 2009; Roza et al., 2019). Second, the accumulated LPA promotes the release of autotaxin (ATX) from astrocytes, further enhancing the glutamate concentration in the synaptic cleft (Thalman et al., 2018). Other evidence is also available showing that PRG-1 modulates LPA release in a non-cell-autonomous pattern, thus leading to an elevation of pre-synaptical glutamate vesicle release, as well as a series of alterations in structural plasticity such as filopodia formation, neurite extension, and brain reorganization after lesion (Broggini et al., 2010; Coiro et al., 2014).

### LPA and Modulation of Glial Cell Activity

LPA modulates the activity of neural glial cells through three actions: (1) LPA mediates the development of oligodendrocytes; (2) LPA regulates the activation of microglia and plays a crucial role in modulating neuroinflammation; and (3) LPA interacts with astrocytes and induces neuronal differentiation.

Accumulating evidence has proposed the involvement of LPA in oligodendrocyte development. Studies on neonatal Lpar1−/− mice have revealed that a deficiency of the LPA1 gene in adult mice could lead to a decrease in myelination and oligodendrocyte survival, suggesting a pro-differentiation role LPA1 on developing and mature oligodendrocytes (Weiner et al., 1998; Choi and Chun, 2013; García-Díaz et al., 2015). Furthermore, ATX has been shown to reduce the adhesion between oligodendrocytes and extracellular matrix molecules, such as fibronectin, laminin 2, and vitronectin through the ATX-LPA-G_α i_ pathway in CNS development (Fox et al., 2003; Nogaroli et al., 2009). Besides, ATX has also been demonstrated to modulate oligodendrocyte development indirectly. Clair et al. (2003) observed that ATX was capable of catalyzing S1P formation, which has been considered as a crucial bioactive factor, which regulates oligodendrocyte maturation and function (Jung et al., 2007; Cui et al., 2014).

Several years of hard work have expanded our knowledge of microglia and their role as a resident immune regulator in the brain. Microglia have been found to produce inflammatory factors in the CNS as well as modulate inflammation and repair. It has been demonstrated that ATX acts as a negative regulator during oxidative stress by inhibiting microglia activation and subsequent release of pro-inflammatory mediators, TNF-α and IL-6, through the LPA-LPA1-ERK1/2 pathway (Kwon et al., 2018). Meanwhile, another LPA pathway has been found to drive microglia towards a pro-inflammatory phenotype. Plastira et al. (2017) demonstrated that LPA could enhance the migratory capacity of microglia, increase the secretion of pro-inflammatory cytokines, as well as produce reactive oxygen species (ROS) and NO to exert cytotoxicity towards neurons. These effects were proven to be mediated through the LPA-LPA5-PKD pathway. Interfering with this axis leads to a reduction in microglial migration and pro-inflammatory cytokine secretion, thus abrogating the LPA-induced neuroinflammation (Plastira et al., 2017). Other research has indicated that LPA can promote BV-2 (expressing LPARs 2, 3, 5, and 6) and primary murine microglia (PMM, expressing LPARs 1, 2, 4, 5, and 6) to transform towards a pro-inflammatory M1-like phenotype. Furthermore, inhibition of microglia with pharmacological LPA5 antagonist TCLPA5 disrupted most of the pro-inflammatory effects, indicating the potential regulatory role of the LPA-LPA5 signaling axis in activating microglia-mediated neuroinflammation (Plastira et al., 2016). Besides the mentioned effects, ATX has also been suggested to function in both acute and chronic inflammatory conditions, while more involvement of ATX-mediated pro-inflammatory response was detected in chronic inflammation (Swaney et al., 2010).

In addition to the above-mentioned roles of LPA in regulating oligodendrocyte development and microglia-mediated inflammation, LPA has also been suggested to stimulate astrocyte-induced neuronal communication (Shano et al., 2008; García-Díaz et al., 2015). Spohr et al. (2008) have proposed an underlying mechanism by which neurons and astrocytes communicate. They have indicated that LPA plays a role in affecting cellular events such as neurite outgrowth, cell fate commitment, and maturation. These cellular responses are mediated by LPA induced rearrangement of fibronectin and laminin through the LPA1/2-EGF-MAPK-fibronectin/laminin pathway as well as the LPA4-PKA-fibronectin/laminin pathway (Spohr et al., 2008; E Spohr et al., 2011). Also, other cellular responses such as neuronal differentiation require the participants of LPA signaling, as it has been shown that the generation of nerve growth factor (NGF) by astrocytes can be increased by LPA (Furukawa et al., 2007).

## Crosstalk Between LPA and The Pathogenesis of Alzheimer’s Disease

For several years, AD has been clinically characterized by progressive dementia with aberrant pathological deposits of intracellular NFTs and aggregation of extracellular Aβ peptides. Recent etiological studies of AD have focused on the vascular factors which participate in neuronal degeneration (Greenberg et al., 2020). In particular, oxidized low-density lipoprotein (oxLDL) has been demonstrated to contribute to AD pathology by regulating Aβ generation (Sun et al., 2003). As the primary component of oxLDL, LPA has been suggested to disrupt the normal functions of the blood-brain barrier and lead to AD-related cellular dysfunction (Frisardi et al., 2011).

**Figure 1:**
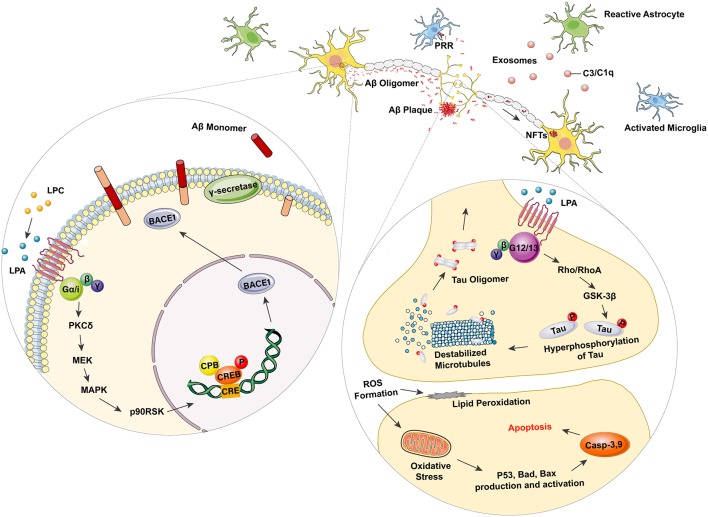
Lysophosphatidic acid (LPA) as a promoter in Alzheimer’s disease (AD) related neurodegeneration. LPA can elevate the binding activity of cAMP response element-binding protein (CREB) with β-site of amyloid precursor protein (APP)-cleaving enzyme (β-secretase, BACE 1) promoter at the CRE site through the G_α/i_-PKCδ–MEK–MAPK–p90RSK–CREB signaling pathway. Overexpressed BACE 1 with γ-secretase then acts as the processing enzymes to cleave APP and produce Aβ monomers, thus leading to the dynamic polymerization and depolymerization of Aβ, as well as the AD-related pathology. Excessive activation of LPA mediated G_α12/13_-RhoA/Rock-GSK3β pathway also results in the hyperphosphorylation of Tau, as well as the subsequent disorganization of microtubule (MT). The hyperphosphorylated Tau protein can be further polymerized to form intracellular NFT deposits, which are considered as another hallmark of AD. Moreover, microglia are also activated in AD-related pathogenesis. Astrocytes become stimulated and transform into their A1 phase. The active A1 astrocytes promote the secretion of complement-contained exosomes to mediate neurological damage *via* the complement pathway. Microglia combines with toxic amyloid-β (Aβ) oligomer and fibrils through pattern recognition receptors (PRRs) and initiates the release of pro-inflammatory mediators, such as iNOS, TNF-α, IL-1β, and IL-6.

Decades before the occurrence of dementia symptoms, senile plaque (SP) deposits containing Aβ peptides are a pathological hallmark in the brains of AD patients. Excessive aggregation of Aβ peptides has been ascribed to the imbalance between Aβ production and clearance. Sequential cleavage of amyloid precursor protein (APP) by its processing enzymes, β-secretase (β-site of APP-cleaving enzyme, BACE1) and γ-secretase (presenilin subunits PS1 or PS2), has been suggested as the main contributor to Aβ-induced AD pathology (Tiwari et al., 2019). Several lines of evidence have suggested that LPA plays a role in Aβ production. Shi et al observed that LPA could significantly upregulate the binding activity of CREB (cAMP response element-binding protein) by utilizing the BACE1 promoter at the CRE site. This work thus increased the CREB-mediated β-secretase (BACE1) expression without altering the expression level of APP or γ-secretase complex (Shi et al., 2013). This alleged role of LPA in promoting Aβ production was later shown to be mediated through the PKCδ–MEK–MAPK–p90RSK–CREB signaling cascade (Shi et al., 2013; Figure 1).

In addition to the Aβ-associated pathogenesis, the function of phosphorylated tau in forming intracellular NFTs, has been considered as another key promoter in AD pathology. As the key element in forming microtubule-associated proteins (MAPs), tau has been validated to play a crucial role in microtube assembly as well as maintaining axonal homeostasis (Evans et al., 2000). As mentioned in earlier sections, LPA has been demonstrated to affect the GSK3β-mediated interactions between microfilaments (MF) and polymerization-dependent microtubule (MT), thus promoting microtubule depolymerization, neurite extension and axon differentiation (Fukushima et al., 2002a,b, 2011; Fukushima and Morita, 2006). Under pathological circumstances, however, the finely tuned MF-MT dynamics, which require sophisticated regulation through the LPA-mediated Gα_12/13_-RhoA/Rock-GSK3β pathway, is altered in AD (Sayas et al., 1999, 2002b). Additional studies have shown redundant neurite retraction in neurodegeneration (Sayas et al., 2002a), suggesting excessive activation of GSK3β induced by LPA. By using the GSK-3 inhibitor lithium, Ramesh et al. (2018) have also identified the involvement of GSK-3β induced tau hyperphosphorylation. These results have demonstrated a dual modulatory role of LPA in regulating MT dynamics. In physiological conditions, the equilibrium of MT depolymerization is shown to promote neurite outgrowth, while pathological MT depolymerization and tau hyperphosphorylation leads to excessive NFT deposits (Figure 1).

Another important pathological feature in AD is the activation of glial cells, particularly the activation of astrocytes and microglia in the CNS. Astrocytes are a type of glial cell that acts as key regulators in neurotransmission, calcium homeostasis, and the formation, maturation, and elimination of synapses. Astrocytes have also been demonstrated to provide nutritional and trophic support for neurons. Several lines of evidence have identified morphological changes of astrocytes in a range of CNS disorders. These alterations, including astroglial atrophy and reactive astrogliosis, are closely related to the pathological response of astrocytes in AD pathology. Collective evidence has indicated that reactive astrocytes can promote the inflammatory processes observed in AD. Reactive astrocytes have been found near to activated microglia, surrounding SPs in the neurodegenerative regions of post-mortem AD brains (Olabarria et al., 2010; Liddelow et al., 2017). Astroglial atrophy was also identified in AD models, as progressive cognitive deterioration caused by the decrease in astrocytic arborization and related loss of synaptic connectivity was also detected (Kulijewicz-Nawrot et al., 2012). Microglia are a cluster of resident immune cells in the CNS which function in maintaining brain homeostasis as well as the plasticity of neural circuits (Ji et al., 2013). There are numerous highly conserved pattern recognition receptor (PRR) expressing in microglia, such as the toll-like receptors (TLRs), nucleotide-binding oligomerization domain-like receptors (NOD-like receptors, NLRs), scavenger receptors (SRs) and receptors for advanced glycation end products (RAGEs; Parkhurst et al., 2013; Dansokho and Heneka, 2018). These PRRs then recognize exogenous signals (such as pathogen-associated molecular patterns, PAMPs) as well as self-derived signals (such as danger-associated molecular patterns, DAMPs) to activate microglia and generate inflammatory mediators (Pereira et al., 2019). Evidence is available showing that microglia function as promoting factors rather than a relevant feature in AD pathogenesis (Hansen et al., 2018). Zhang et al. (2016) found that most of the AD-related risk genes were preferentially expressed in microglia rather than any other cell types in the brain. This is in line with the results demonstrated by several emission tomography (PET) studies, which identified that the activation of microglia was positively correlated to Aβ deposits, tau aggregation and cognitive deficiency in MCI and AD patients (Hamelin et al., 2016; Dani et al., 2018). Similar histopathological research also suggested the co-localization of activated microglia with Aβ aggregation as well as tau oligomers in AD brains (Zotova et al., 2013; Nilson et al., 2017). The activation of microglia has been widely recognized as a major contributor to neuroinflammation (Heneka et al., 2015a), it has also been verified that Aβ plaques and NFTs were both invariably correlated with microglia-induced neuroinflammation (Pereira et al., 2019). Several studies have proposed a plausible mechanism of microglial activation, which is initiated by interaction with toxic Aβ oligomers and fibrils through PRRs, such as NLRP3, NLRP1, CD36, CD14, TLR2, RAGE, thus inducing the release of pro-inflammatory mediators like iNOS, TNF-α, IL-1β and, IL-6, as well as the elevated expression of cell adhesion markers CD11b and CD68 (Fassbender et al., 2004; Halle et al., 2008; Jana et al., 2008; Stewart et al., 2010; Venegas and Heneka, 2017). Similar results have also demonstrated that Aβ peptides and fibrils can act as disease-associated molecular patterns (DAMPs). This, in turn, leads to activation of Toll-like receptors (TRLs) and the NRLP3 inflammasome, leading to the generation of TNFα, IL-1β, and other pro-inflammatory mediators by microglia (Heneka et al., 2013; Heneka et al., 2015b). Evidence is also available showing that tau oligomers, as well as NFTs, can induce the activation of microglia and generation of NO and IL-6 (Morales et al., 2013). In addition to the outlined evidence, it is also well-illustrated that microglia and astrocytes can act as co-contributors to promote neuroinflammation. It has been shown that activated microglia release a great range of pro-inflammatory mediators, such as IL-1α, TNF-α, and C1q, and trigger the transformation of astrocytes into a neurotoxic “A1” reactive state, in which astrocytes lose the function of maintaining the survival of neurons and oligodendrocytes (Liddelow et al., 2017). Moreover, Goetzl et al. (2018) have also demonstrated an elevated level of complement factors, such as C3 and C1q in astrocyte-derived exosomes from AD patients, supporting the finding that “A1” reactive astrocytes induce AD pathogenesis by promoting the secretion of complement proteins (Goetzl et al., 2018; Figure 1).

## Physiological Roles of S1P in The Nervous System

As mentioned in earlier sections, the generation of S1P is under precise modulation by two evolutionarily conserved lipid kinases known as the sphingosine kinase-1 (SphK1) and sphingosine kinase-2 (SphK2). The subcellular localization, as well as the biological effect of S1P, have been suggested to be determined by the two isoenzymes (Spiegel and Milstien, 2003; Chan and Pitson, 2013; Santos and Lynch, 2015). It has been demonstrated that SphK1 activation requires the re-localization from the cytoplasm to the plasma membrane (Neubauer and Pitson, 2013). Furthermore, SphK1 has been shown to mediate S1P induced effects such as cell proliferation, migration, and survival (Zhu et al., 2010; Chan and Pitson, 2013; Gassowska et al., 2014). However, another lipid kinase SphK2 has been proved to localize mainly in cellular organelles, such as the nucleus and ER, and the downstream intracellular functions mediated by SphK2 induced S1P are yet more complicated (Neubauer and Pitson, 2013). It has been shown that the SphK2 mediated S1P generation can also function as a pro-survival factor as well as a cellular apoptosis contributor without activating S1PRs (Pyne and Pyne, 2010). In this part, the S1P-mediated biological functions in the CNS will be discussed in detail.

### S1P and Brain Development

In the decades following the discovery of S1P, it was regarded as an inactive end product of sphingosine metabolism. Recently immense amounts of research have now indicated the crucial role of S1P in regulating intracellular signaling transduction as an active lipid metabolite (Ghasemi et al., 2016). Both SphK1 and SphK2 have been identified as being expressed in the CNS. Studies have shown that the deficiency of either enzyme would not cause obvious CNS phenotype changes, while the deletion of both Sphk1 and Sphk2 in animals can lead to severe developmental brain defects (Mizugishi et al., 2005; Ghasemi et al., 2016). As demonstrated by Mizugishi et al. (2005), mice lacking both Sphk1 and Sphk2 can result in a deficiency of S1P production, thus leading to severe abnormalities in neurogenesis as well as angiogenesis, and ultimately impair brain development. There is also evidence showing that the single deletion of Sphk2, which leads to a reduction of nuclear S1P level, can induce abnormalities in spatial memory and fear extinction (Hait et al., 2014). These authors believed that the downregulation of Sphk2 could impair S1P-mediated endogenous inhibition of histone deacetylase (HDAC), thus decreasing histone acetylation as well as depressing the expression of cognitive related genes (Hait et al., 2014).

S1P functions in modulating brain development by binding to the widely expressed S1P receptors in a range of CNS cells, including oligodendrocytes, neurons, astrocytes and neurogenic microglia (Anelli et al., 2005; Jaillard et al., 2005; Satoh et al., 2008). Evidence demonstrates that the expression profile of S1P receptors is dynamic, changing depending on the developmental stage and the certain physiological function they exert (Jaillard et al., 2005). Moreover, growth factors have also been validated to act as modulators in determining the expression of receptors and the activation of cellular responses. For example Jung et al. (2007) have shown that platelet-derived growth factor (PDGF) can trigger the down-regulation of oligodendrocyte progenitor cells (OPCs)-mediated S1P5 expression as well as the up-regulation of S1P1, S1P2 and S1P3 expression in these cells. Although it has been demonstrated that the widely expressed S1P receptors are associated with the signaling pathways required for maintaining brain functions, the cells that express S1P receptors are mainly glial rather than neuronal. Several studies have suggested a high expression of S1P receptors (S1P3 > S1P1 > S1P2) in astrocytes, while the expression of S1P5 is relatively low (Rao et al., 2003; Anelli et al., 2005). In oligodendrocytes, however, the expression of S1P receptors presented a distinct profile among different subtypes, as S1P5 demonstrated a relatively high expression compared to the other S1P receptors (S1P1 = S1P2 > S1P3; Yu et al., 2004). Accordingly, various cellular responses have been validated to be induced by the activation of different S1P receptor subtypes (Jung et al., 2007). Novgorodov et al. (2007) have verified that S1P can inhibit the migration of OPCs by binding to S1P5, while it has also been demonstrated to promote the differentiation of oligodendrocytes through other S1P receptor subtypes (Novgorodov et al., 2007; Wang and Bieberich, 2018). Similar results have also demonstrated that S1P can interact with S1P receptors and induce the migration of neural stem progenitor cells (NSPCs) to the sites of injury (Kimura et al., 2007). Other evidence has also indicated that S1P-induced cellular responses are cell types specific, as S1P was shown to mediate neurite contraction and soma rounding in N1E-115 neuronal cells, which can not be observed in PC12 cells (Postma et al., 1996; Edsall et al., 1997).

### S1P as a Neuroprotective Factor

Several lines of evidence have identified that oxidative stress leads to a range of cytopathic events, and therefore, leads to cell apoptosis in both mitochondrial-dependent as well as mitochondrial-independent ways (Sinha et al., 2013). Evidence shows that the impact of oxidative stress on cell fate is bilateral. It has been suggested that high levels of ROS can induce the activation of sphingomyelinase (SMase) and shift the “sphingolipid rheostat” towards ceramide accumulation, thus triggering cell apoptosis (Petrache and Berdyshev, 2016). Meanwhile, temperate oxidative stress can activate SphK1 and lead to the upregulation of S1P, thereby promoting cell survival (Martín-Montañez et al., 2019). The protective role of S1P against oxidative stress-induced cellular damage has been validated, as proven by Pyszko and Strosznajder that S1P treatment can reverse MPP+ mediated free radical production and cellular apoptosis (Pyszko and Strosznajder, 2014). Similar results have also demonstrated that S1P can activate the Akt pathway and inhibit the JNK pathway to alleviate H_2_O_2_-mediated growth arrest (Lee et al., 2012). Furthermore, it has been suggested that S1P could act as an antioxidant factor by enhancing the activity of superoxide dismutase and catalase (Chawla et al., 2014).

**Figure 2:**
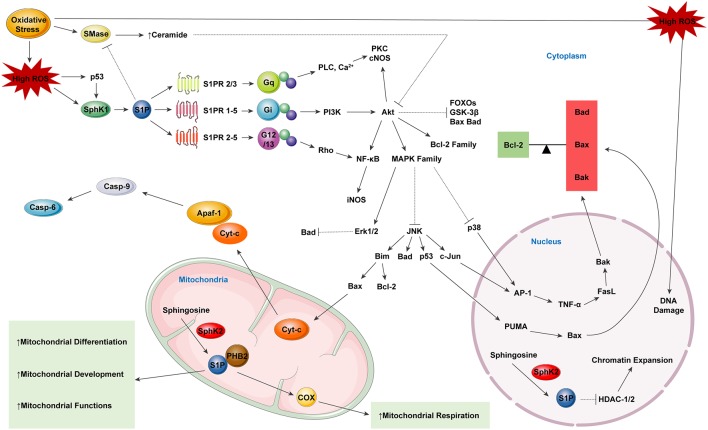
Sphingosine-1-phosphate (S1P) as a neuroprotector, modulating reactive oxygen species (ROS) induced cell apoptosis. S1P serves in an indirect way of protecting neural function in neurodegeneration. By binding to specific S1PRs, S1P in the cytoplasm can modulate the balance between the pro-apoptotic factors Bad, Bax, and Bak as well as the anti-apoptotic mediator Bcl-2. The equilibrium between the factors of the two major categories determine the cell fate. Besides, S1P produced in mitochondria can specifically bind to prohibitin 2 (PHB2), which in combination with cytochrome-c (Cyt-c), helps to stabilize mitochondrial assembly. S1P can also promote the differentiation, development, respiration and biological functions of mitochondria.

In line with the above-mentioned roles of S1P in protecting cells from oxidative stress, there are also several studies showing that S1P can act as an inhibitor of neuronal apoptosis (Edsall et al., 1997). S1P has been shown to exert anti-apoptotic effects by inhibiting oxidative stress (Ghasemi et al., 2016). S1P has also been demonstrated to prevent apoptosis by blocking Aβ toxicity (Edsall et al., 1997; Gomez-Brouchet et al., 2007). Studies have suggested that S1P may also play a two-sided role in modulating cell apoptosis since transient S1P exposure is neuroprotective, while critically high levels of S1P are neurotoxic and can trigger neuronal apoptosis (Hagen et al., 2009; van Echten-Deckert et al., 2014; Ghasemi et al., 2016). However, Hagen et al. (2009) have demonstrated that only SphK2-mediated production of S1P can lead to apoptosis, indicating the role of subcellular localization of S1P in determining its biological function. Moreover, the key molecules in S1P-related signalings, such as the AP-1, ERK, or NF-κB, also play an ambiguous role in regulating cell apoptosis, thus exerting a dual effect on cell fate (Oh et al., 2017; Park et al., 2018). One of the most important signaling pathways related to S1P-mediated anti-apoptosis is the S1PR- Jnk/p38/ERK-AP-1 cascade (O’Neill et al., 2011; Jazvinšćak Jembrek et al., 2015). S1P exerts its anti-apoptotic role mainly by activating p38 and ERK, as well as inhibiting Jnk, thus triggering the downstream inhibition of transcription activator AP-1 in the nucleus, thus preventing cell death (Hasegawa et al., 2010; Van Brocklyn and Williams, 2012). Another crucial anti-apoptotic signaling pathway that is activated by S1P is the S1PR-PI3K-Akt-Bad/GSK-3β/FOXOs pathway, the disruption of which can lead to severe AD pathogenesis (Moloney et al., 2010; Safarian et al., 2015). S1P has been shown to suppress the activation of tau kinase GSK-3β as well as inhibit the pro-apoptotic proteins Bad, FOXO1a, 3a, 4, and 6 *via* PI3K-Akt, functioning to protect cells from apoptosis (Santos and Lynch, 2015; Maiese, 2017). Other mediators including transcription factor NF-κB and HDACs HDAC1 and HDAC2 are also under the regulation of S1P related SL signaling (Dai et al., 2005; Hait et al., 2009). Also, S1P has been shown to inhibit acid sphingomyelinase (aSMase) mediated ceramide production, thus suppressing ceramide-induced cell apoptosis (Jazvinšćak Jembrek et al., 2015; Figure 2).

Mitochondria have been considered to play a crucial role in maintaining metabolic homeostasis, regulating apoptotic processes and oxidative energy metabolism, as well as modulating ROS production (Lou et al., 2020). SphK2 has also been found to be expressed in mitochondria in addition to being expressed in the nucleus, and evidence has suggested that SphK2 may function in the production of S1P in mitochondria (Chan and Pitson, 2013; Santos and Lynch, 2015; Ghasemi et al., 2016). Several lines of evidence have indicated a protective function of SphK2-induced S1P in mitochondrial homeostasis. As shown by Strub et al. (2011), S1P that is produced within mitochondria can specifically bind to prohibitin 2 (PHB2), which functions in conjunction with cytochrome c oxidase (complex V) in mitochondria and stabilizes its assembly. It has been demonstrated that SphK2 deficiency can lead to mitochondrial respiratory dysfunction and subsequent energy metabolism disorders, which can be restored by exogenous S1P treatment (Strub et al., 2011). Furthermore, S1P has been proved to promote mitochondrial biogenesis by increasing mitochondrial DNA replication, transcription and mitochondrial mass (Shen et al., 2014). There is also evidence showing that S1P treatment can alleviate oxygen-glucose deprivation (OGD) induced inner membrane depolarization, which can induce the formation of MMP (mitochondrial membrane permeability) and directly affect mitochondrial function (Agudo-López et al., 2010). S1P has been shown to play an indirect role in protecting mitochondrial function by modulating the SL rheostat. Accordingly, S1P can inhibit sphingomyelinase (SMase) enzyme activity as well as the subsequent production of ceramide, thus altering the ceramide-induced mobilization of ER Ca^2+^, the activity of mitochondrial respiratory complexes I and III, and also forming channels in the outer mitochondrial membrane (OMM; Kim and Sieburth, 2018; Oleinik et al., 2019).

Autophagy plays an important physiological role in the survival of cells with high energy demands such as neurons (Menzies et al., 2015). While normal levels of autophagy act as a cellular survival and adaptation program that is essential for maintaining cell homeostasis under different stress conditions, excessive autophagy can lead to autophagic cell death (Mariño et al., 2014). S1P, as one of the important bioactive SL metabolites, has been shown to regulate cellular apoptosis as well as autophagy (Wang and Bieberich, 2018). Recent studies have focused on the underlying mechanisms by which S1P-associated autophagic pathways might influence neurodegeneration (Wang and Bieberich, 2018). Several lines of evidence have indicated that the dynamic equilibrium between the formation and degradation of S1P by three enzymes (SphKs, which catalyze S1P formation, S1P phosphatases (SGPPs) and sphingosine phosphate lyase 1 (SGPL1), which catalyze S1P degradation) are of vital importance for S1P-related autophagic processes (Spiegel and Milstien, 2003; Lépine et al., 2011; Moruno Manchon et al., 2015). It has been demonstrated that SphK-1 and S1P can induce autophagy in nutrient deprivation conditions to protect cells from apoptosis (Lavieu et al., 2006). Also, similar results have been shown in primary neurons, as cytosolic S1P has been identified to modulate neuronal autophagy (Lépine et al., 2011; Moruno Manchon et al., 2015). Sheng et al. (2014) have also demonstrated that SphK2 can mediate hypoxic preconditioning-induced autophagy, and found that pretreatment with SphK2 inhibitors prevented S1P-induced apoptosis. It has been proposed that S1P may induce autophagy by inhibiting the mTOR pathway which could be considered as a potential underlying mechanism for autophagy inhibition (Orsini et al., 2019). In line with the previous studies, it has also been suggested that S1P could promote cell autophagy, as inhibition of SphK-1 with SK1-I increased autophagic flux and cell apoptosis (Lima et al., 2018). However, recent studies have demonstrated that SPHK1 deletion leads to upregulated autophagic flux in primary embryonic fibroblasts of mice, indicating an inhibitory role of SphKs in autophagy (Young et al., 2016). Moreover, several lines of evidence have suggested an indirect pathway by which S1P could modulate cell autophagy, as the S1P degradation product, phosphatidylethanolamine (PE), has been observed to result in deficient autophagy (Fyrst and Saba, 2008; Mitroi et al., 2017).

### S1P and Neurotrophic Factors

It has been widely demonstrated that S1P interacts with various NGFsto promote cell development and survival (Ghasemi et al., 2016). Yamagata et al. (2003) have observed that S1P mediates the expression and release of glial-derived neurotrophic factor (GDNF) in cortical astrocytes, which can subsequently induce the growth and proliferation of these cells. Similar results have indicated that the S1P receptor agonist fingolimod phosphate (FTY720-P) can elevate the expression level of brain-derived neurotrophic factor (BDNF) in neurons, thus mediating a protective effect against oligomeric amyloid β-induced neurotoxicity (Doi et al., 2013). In line with these findings, FTY720-P has also been proved to induce the production of neurotrophic mediators, such as LIF, HBEGF, and IL-11 in astrocytes, as well as inhibit the expression of TNF-induced inflammatory genes (Brinkmann et al., 2002; Hoffmann et al., 2015). Furthermore, S1P is shown to mediate NGF-induced neurofilament expression and cell survival, while inhibition of Sphk activity suppressed NGF mediated cell differentiation (Edsall et al., 1997). Moreover, Hall et al. (1988) have identified the S1P decrease, that resulted from the shifting of the “sphingolipid rheostat” towards ceramide, could inhibit NGF-induced neurite outgrowth. In addition to the mentioned roles of S1P in modulating NGF-mediated biological response, it has also been demonstrated that NGF can, in turn, stimulate Sphk1 through TrkA signaling to elevate S1P expression, thus activating S1P1 and S1P2 to promote neurite extension (Toman et al., 2004). Similar results have also demonstrated that the GDNF can increase the expression of SphK1, thereby promoting the production and secretion of S1P in a neuroblastoma cell line (TGW; Murakami et al., 2011).

### S1P and Neurotransmitter

Several lines of evidence have indicated the role of S1P in regulating neurotransmitter release as well as neuronal excitability in neural tissues, among which regulation mainly involves modulation of epinephrine and glutamate synaptic transmission. Recent studies have demonstrated that S1P is involved in bortezomib-induced neuropathic pain by increasing presynaptic glutamate release; this effect was later shown to be mediated by S1PR signaling (Sheng et al., 2014; Boyette-Davis et al., 2015; shown in Table 1). In addition to this, there is a wealth of evidence indicating that exogenous, as well as endogenous, S1P can stimulate glutamate release from the excitatory synaptic terminals (Kajimoto et al., 2007). It has also been shown that the S1P-induced hippocampal glutamate release is exclusively mediated by S1P3 (Kajimoto et al., 2007; Kanno and Nishizaki, 2011). This is in line with the evidence showing that *in vitro* treatment of Sphk and S1P in hippocampal slices or cultures can demonstrate increased glutamate release from synaptosomes as well as an elevated rate of spontaneous glutamate transmission (Darios et al., 2009). Furthermore, S1P is also validated to mediate glutamate release through an intrinsic pathway, as deletion of SphK-1 can lead to a decrease in activated synaptic glutamate transmitters, thereby impairing the long term-potentiation as well as causing cognitive dysfunction (Kajimoto et al., 2007; Chan and Sieburth, 2012). Schenk et al. (2005) have proposed that S1P induced endogenous glutamate release may function in activating presynaptic AMPA receptors to mediate the dispersion of SynI, which plays a crucial role in modulating exocytosis. In contrast, it has also been suggested that S1P may inhibit glutamate transmission through S1P1 signaling in cortical regions (Sim-Selley et al., 2009), which suggests that S1P may function in a region-specific way to modulate glutamatergic neurotransmission (Welch et al., 2012). In addition to this, a role for S1P in depolarization mediated release of noradrenaline has also been indicated (Alemany et al., 2001).

## Crosstalk Between S1P and The Pathogenesis of Alzheimer’s Disease

Alterations in SL metabolism, as well as composition, have been considered as crucial contributors to neurodegeneration (Wang and Bieberich, 2018). AD-related lesions including synaptic loss, aberrant Aβ aggregations, NFTs, gliosis, and neuroinflammation have all been demonstrated to promote cerebral atrophy and cognitive impairment (van der Kant et al., 2020). It has been shown that S1P participates in a vast range of pathological conditions such as cancer, autoimmunity, cardiovascular diseases and diabetes (Proia and Hla, 2015), and recent studies have also indicated a key role of S1P in neurodegeneration (Chakrabarti et al., 2016; Karunakaran and van Echten-Deckert, 2017). Abnormal SL metabolism was initially observed in brain samples from AD patients when compared to age-matched normal individuals, with the S1P level being found to decrease with elevated ceramide expression (He et al., 2010). This is in line with cellular studies suggesting that Aβ treatment could drive the “sphingolipid rheostat” towards ceramide and lead to a reduction in S1P level of neuronal and glial cells (Lee et al., 2004; Gomez-Brouchet et al., 2007). Recent studies have identified a decreased level of SphK1 and S1P coupled with elevated SPL in the entorhinal cortex of AD patients, further illustrating the deregulation of SL metabolism in AD pathogenesis (Ceccom et al., 2014). SphK1 overexpression has been identified to switch the “sphingolipid rheostat” toward S1P production, thus protecting the neurons against Aβ-induced neurotoxicity (Yang et al., 2014). This effect was later shown to be mediated by the S1P-induced inhibition of acid sphingomyelinase (aSMase) activation as well as the subsequent ceramide production (Claus et al., 2009; Justice et al., 2018). In line with these observations, He et al. (2010) have identified decreased S1P expression with elevated Aβ peptides and phosphorylated tau protein in AD brains. Besides, the authors showed that *in vitro* treatment of neuronal cells with Aβ induced S1P inhibition and neuronal apoptosis (He et al., 2010).

In contrast to the identified reduction of SphK1 expression, the role of SphK2 in AD brains remains controversial. Several lines of evidence have suggested that there is an increase in SphK2 activity in the frontal cortex while SphK2 expression inhibition was observed in the temporal cortex and hippocampus of AD brains (Takasugi et al., 2011; Maceyka et al., 2012; Asle-Rousta et al., 2013). Dominguez et al. (2018) have demonstrated the re-localization of SphK2 from the cytosol to the nucleus relative to Aβ pathology, leading to the upregulation of intranuclear S1P expression as well as a series of deleterious responses. This is following the evidence that S1P functions by indirectly modulating β-site APP cleaving enzyme-1 (β-secretase, BACE1), which is considered as the rate-limiting enzyme for amyloid-β peptide (Aβ) production (Takasugi et al., 2011; Maceyka et al., 2012). Since SphK2 and S1P have been indicated to regulate the expression of HDAC1/2, there is also evidence suggesting that S1P acts as an underlying epigenetic regulator in AD-related cognitive dysfunction. Graff et al. (2012) have demonstrated in their study that elevated levels of HDAC2 could epigenetically repress the synaptic genes that related to cognitive deficits, while Panikker et al. (2018) suggested that decreasing HDAC2 levels in AD-related APP brain could reverse the neuroepigenetic changes in activating synaptic plasticity genes, as well as restoring brain morphology and cognition (Graff et al., 2012; Panikker et al., 2018).

## Conclusion

LPLs are a cluster of bioactive intermediates, among which the well-characterized LPA and S1P have been identified as playing a crucial role in a series of cellular responses such as neurogenesis, proliferation, survival, cytoskeleton remodeling, morphological changes, migration and differentiation through the G protein-coupled receptor signaling. Several years of research have identified the importance of well-regulated LPA and S1P metabolism in maintaining neuronal health as an integrative process. Dysfunction of the regulating circuits of the two best-characterized signaling lipids has been verified to be a participant in a range of pathological processes, especially the neurodegeneration. Herein, we have demonstrated plausible mechanisms by which LPA and S1P modulate AD-related pathologies. Meanwhile more specific studies are still needed to explore the remaining questions such as as: (1) what are the exact functional roles of LPA and S1P in modulating physiological responses in the CNS? (2) Is LPL disruption the primary initiator in promoting AD pathogenesis? (3) How do LPLs work in an integrative mechanism to promote AD development? and (4) Is there any way to prevent LPL-related neurodegeneration? Due to the wide distributions of LPLs and their signaling pathways in CNS, a wide range of work is required in the future to illustrate their physiological activities.

## Author Contributions

YH and MC conceptualized the manuscript. YH wrote the initial draft. YH, MG, YF, QD and MC contributed substantially to discussions of the article content and review and/or editing of the manuscript before submission.

## Conflict of Interest

The authors declare that the research was conducted in the absence of any commercial or financial relationships that could be construed as a potential conflict of interest.
